# Charity

**Published:** 1893-09

**Authors:** W. S. Curtis

**Affiliations:** West Randolph


### Charity.

BY DR. W. S. CURTIS, WEST RANDOLPH.

Read before the Vermont State Dental Society, March 18th, 1893.

This subject is a broad and far-reaching one and has ever been an element of progress in the affairs of this world, without which we should be below the animal; it being manifest in different degrees in nearly all forms of animal life.

Charity forms the basis of all things taught by Christ, without which all is vanity and nothing remains.

It is the foundation of all so-called Christian work in all civilized lands, and for which much time and many millions are given yearly. It has inspired more men and women to noble deeds than all other elements combined.

The motive power of all reforms the world has ever seen, or ever will see, is this same Charity.

Does it seem fitting that an element so closely allied to all that is grand and noble should be lost sight of by any intelligent body of men in any civilized land?

I do not wish to cast any reflection upon our profession as being an uncharitable body, or that they are more so than other intelligent organizations within our borders, but I do think we are much less charitable than we might be, and less than we would be, if we as a body, would spend a few moments, from time to time, in thinking into existence some benevolent system by which our patients and ourselves would receive much benefit.

It must be conceded that the united effort of the dental profession would be a power behind any movement. Therefore, it is the purpose of this paper to introduce an idea which, though possibly not new to all, has never been agitated, to my knowledge, by any member of our profession.

It is a well-known and lamentable fact to most of us, who have been in practice for any length of time, that a very large number of children and young people within the immediate vicinity of every dentist need more or less of our services but who are too poor to obtain them; therefore, they must do without; adopt the credit system and they seldom pay; or the operator must contribute more or less of his otherwise valuable time for the relief of their necessities; either of which is all wrong and makes more or less evil to both operator and patient.

Unfortunately very few of us are financially able to contribute much time to charity patients, although many desire to do so, and in an effort to satisfy that desire do much more in that line than is for their own material good, and to the injury also of all patients able to pay, in consuming both time and money that might otherwise be used to promote the progress and intellectual growth of the dentist to the direct benefit of himself and his patients.

To the end that this evil be remedied by the introduction of some proper system I would suggest, for the consideration of this society and the dental profession in general, that a Dental Benevolent Association be organized in every State that has a dental society, that it be clothed with proper powers granted by the legislature of the various States in which such societies exist, that it shall seek to create a fund by asking the State for an annual appropriation, also by soliciting through various channels the contributions of all kindly disposed persons, and many others who possessing more or less money, when called upon to part with their worldly possessions by the stern edict of their Creator, would, no doubt, as they look about for some place to deposit their gold conclude to drop something for the benefit of the masticating organs of the rising generation; that such fund, when created, shall be used for the relief of charity patients in all localities of the State where any member of the association is in practice; either by allotment pro rata to the various towns to be drawn upon by the local dentist performing such services, or by a direct salary paid to various operators appointed by the society who shall travel about and do such work at the various offices where members are located.

Most of us have extra chairs and equipments that we would gladly donate for such work.

Of course, the amount of work done must be governed by the size of the fund, and that no imposition be practiced, some local board of civil authority should determine, through its chairman in council with the local member of the association, who such patients shall be, and determine upon and give a certificate of amount to be done and of what variety, the details of which could be adjusted when the society should determine what method they would adopt for the expenditure of the fund.

I believe if this movement was started in the right spirit, with the combined efforts of the profession behind it, that it would continue to grow until it would lift a heavy burden from the profession in general and confer a lasting benefit upon the rising generation, many of whom would in after years, as they assumed the duties of life remember the benefits they received from the organization and attend to its growth and prosperity.

Among the so-called necessities of life which are recognized as such by the public and for which some provision is now made may be mentioned food, clothing, education, religion, medical attendance and the numerous houses, hospitals and retreats now provided for the various classes of dependents.

All this is the result of love in the human heart, and the amount of effort and money spent in this direction is rapidly increasing. Who of us will attempt to say that the teeth of the rising generation should not have proper care and be classed also among the necessaries of life. And if they should be so classed then the children of to-day have the same moral right to demand that their teeth be cared for, as a necessary requisite for future use and comfort, as they have to demand that some one provide a surgeon to reduce a fracture, which if not done would also deprive them of much that others enjoy.

It is the welfare of the child that is to be considered and in doing so we benefit the future man or woman. It will be claimed, no doubt, that we, as a people, cannot afford to do these things. Suppose all before us had said that, what would be our condition to-day? We are very apt to think that what we have and enjoy are the results of our own efforts. We do not realize that our indebtedness to past generations can only be paid in what we give and leave as a legacy to all who come after us. We labor for others, others are always working for us. We are constantly being "ministered unto." Day by day, year after year, our indebtedness to the community increases. To the community we owe everything. Take the history of our own lives. Think of the millions that have toiled that we might become what we are, an educated, refined and scientific body.

Think of what has been the labor of creating the civilization we unconsciously inherit.

Let us try to realize that our usefulness in this world will be measured by what we add to the inheritance we received for the benefit of our successors.

It is the universal sentiment of all civilized humanity that the necessaries of life be furnished for those who are unable to otherwise obtain them.

And it goes without saying that such provision would generally be made, if it did not cost anything.

It is also conceded by all that any expense which the community incurs that is an actual benefit to the general public is a wise and economical investment. We have only to examine by the light of history the various elements of disintegration now at work upon our social system to realize that the kinship of man, a fundamental principle necessary to progress and happiness, has at last been discovered.

That this fact will eventually result in a more general distribution of our abundant wealth for the public good no student of modern thought can deny.

Shall we not, then, be prepared to absorb our share of it by organizing a benevolent association and, by every honorable means, seek to create a fund and educate the community to realize the necessity of this movement, until at last we should receive the proper recognition and financial support necessary to carry on the work.

If anything can be done to bring about this result is it not desirable to do so? And if done shall we not feel, as we lay down our work and mingle with the silent majority, that this movement was one of many, now being agitated, that was destined to become an important factor in establishing the universal brotherhood of man.

